# Mouse Cytoplasmic Dynein Intermediate Chains: Identification of New Isoforms, Alternative Splicing and Tissue Distribution of Transcripts

**DOI:** 10.1371/journal.pone.0011682

**Published:** 2010-07-21

**Authors:** Anna Kuta, Wenhan Deng, Ali Morsi El-Kadi, Gareth T. Banks, Majid Hafezparast, K. Kevin Pfister, Elizabeth M. C. Fisher

**Affiliations:** 1 Department of Neurodegenerative Disease, UCL Institute of Neurology, London, United Kingdom; 2 Biochemistry and Biomedical Science, School of Life Sciences, University of Sussex, Brighton, United Kingdom; 3 Cell Biology Department, School of Medicine, University of Virginia, Charlottesville, Virginia, United States of America; National Institutes of Health, United States of America

## Abstract

**Background:**

Intracellular transport of cargoes including organelles, vesicles, signalling molecules, protein complexes, and RNAs, is essential for normal function of eukaryotic cells. The cytoplasmic dynein complex is an important motor that moves cargos along microtubule tracks within the cell. In mammals this multiprotein complex includes dynein intermediate chains 1 and 2 which are encoded by two genes, *Dync1i1* and *Dync1i2*. These proteins are involved in dynein cargo binding and dynein complexes with different intermediate chains bind to specific cargoes, although the mechanisms to achieve this are not known. The DYNC1I1 and DYNC1I2 proteins are translated from different splice isoforms, and specific forms of each protein are essential for the function of different dynein complexes in neurons.

**Methodology/Principal Findings:**

Here we have undertaken a systematic survey of the dynein intermediate chain splice isoforms in mouse, basing our study on mRNA expression patterns in a range of tissues, and on bioinformatics analysis of mouse, rat and human genomic and cDNA sequences. We found a complex pattern of alternative splicing of both dynein intermediate chain genes, with maximum complexity in the embryonic and adult nervous system. We have found novel transcripts, including some with orthologues in human and rat, and a new promoter and alternative non-coding exon 1 for *Dync1i2*.

**Conclusions/Significance:**

These data, including the cloned isoforms will be essential for understanding the role of intermediate chains in the cytoplasmic dynein complex, particularly their role in cargo binding within individual tissues including different brain regions.

## Introduction

To transport cargoes within eukaryotic cells, energy dependent motors run along tracks in the cell formed by either the microtubule network (dynein and kinesin motors) or the actin cytoskeleton (used by myosin motors). The motor proteins associated with microtubules can be classified as moving towards the growing ‘plus’ end of microtubules (anterograde transport in axons) or moving towards the ‘minus’ end of microtubules (retrograde transport in axons). While several kinesin motors are responsible for anterograde transport, the cytoplasmic dynein complex is the single main retrograde transport motor.

Two cytoplasmic dynein complexes have been identified, of which cytoplasmic dynein 1 is the most abundant in cells, while cytoplasmic dynein 2 takes part in intraflagellar transport [1], [2], [3]. Cytoplasmic dynein 1 is a multisubunit complex of ∼1.5 MDa; in mammals this complex is thought to consist of a homodimer of heavy chains (encoded by a single gene *Dync1h1*); two intermediate chains (encoded by two genes *Dync1i1*, *Dync1i2*); light-intermediate chains (encoded by two genes *Dync1li1*, *Dync1li2*); light chains (thought to be encoded by three gene families, containing six different genes (*Dynlt1*, *Dynlt3*, *Dynlrb1*, *Dynlrb2*, *Dynll1*, *Dynll2*)) [1], [4], [2], [3]. The core of the dynein complex is a homodimer of the heavy chains which binds to microtubules and enables cytoplasmic dynein to move in an ATP dependent manner [5], [6]. The other dynein subunits are thought to associate in the complex as homodimers and to maintain the stability of the complex, to modulate its activity, and to interact with accessory and cargo proteins [7], [8], [3], [9].

The interaction and regulation of cytoplasmic dynein 1 with its cargos is poorly understood; the intermediate chain proteins DYNC1I1 (IC1) and DYNC1I2 (IC2) play roles in cargo binding and are involved in cargo specificity [10], [7]. It is also clear that interactions of the dynein complex with cargoes often requires the presence of the dynactin complex – another large, multisubunit complex that binds to both the intermediate chains of the dynein complex to modulate dynein-cargo interactions, and to microtubules to modulate the movement of dynein [11], [12]. However, IC1 and IC2 may also interact directly with protein cargos including beta-catenin [13], casein kinase II [14], neurofilaments [15], kinesin light chains 1 and 2 [16] and huntingtin [17].

IC1 and IC2 share 69% protein identity and have a molecular weight of approximately 74 kDa [18], [2], [3], and can form homo- or heterodimers in overexpression assays [19]. They interact with the dynein light chains and the p150 subunit of dynactin at the N-terminus and with the heavy chains through WD40 repeats at the intermediate chain C-terminus [20], [2], [3]. Both proteins are expressed in multiple splice isoforms – alternative splicing occurs at their N-terminal regions and tissue- and development-specific isoforms have been described in human, rat and mouse (for brief discussion and original references see [21], [22], [23], [3]. The functional importance of individual splice isoforms is highlighted by the discovery that in rat one dynein complex containing a IC1 isoform, specifically binds nerve growth factor receptor TrkB-containing endosomes in neurons [10], thus this isoform likely confers cargo specificity on the dynein complex and is important for neuronal function. Conversely, dynein complexes containing IC2 are important for transport of TrkA signalling endosomes in PC12 cells [10].

Since this evidence indicates that alternatively spliced intermediate chain isoforms are central to dynein complex function and cargo specificity, we undertook a survey of their expression in a range of mouse tissues, using both RNA studies and bioinformatics analysis including comparison with human and rat data. Here we show (1) new splice isoforms for both genes, (2) a systematic survey of expression of different intermediate chain splice isoforms in a range of adult and embryonic mouse tissues, including different brain regions and spinal cord, (3) and we also identify transcription of *Dync1i2* from a new upstream promoter that results in an alternative non-coding exon 1 and a second set of *Dync1i2* transcripts.

This survey serves as the basis for a thorough functional dissection of the dynein intermediate chains in mouse and their likely roles in binding different cargoes in a cell-specific and development-specific manner, particularly within the nervous system.

## Results

To determine the splice isoform patterns of the mouse dynein intermediate chain 1 and 2 genes, we used a combination of reverse-transcription polymerase chain reactions (RT-PCRs) of mouse RNA, literature searching and bioinformatics analysis of mouse (version mm9), rat (rn4) and human (hg19) data in the UCSC Genome Browser. We note that because different nomenclatures have already been applied to the few previously described mouse and rat dynein intermediate chain isoforms (**Table 1**), for clarity we base our nomenclature on that of Pfister, Vaughan, Vallee, and colleagues who described three rat protein isoforms of DYNC1I1 (IC1A, 1B, 1C) and three isoforms of DYNC1I2 (IC2A, 2B, 2C) [21], [22], [23]. In line with current gene nomenclature rules, we also include a UCSC or Ensembl accession number relating to a ‘reference’ cDNA sequence for each isoform described below.

**Table 1:** Mouse dynein intermediate chain splice isoforms.

Mouse dynein intermediate chain 1 (*Dync1i1*)						
Mouse isoform name	GenBank accession number	UCSC and/or ENSEMBL accession number for ‘reference sequence’ for each isoform	Homologous published rat isoforms [21], [22] and previously described mouse isoforms, in italics [34]	Exons	Exon 4 alter-native splice site	Number of amino acids
Dync1i1.A		ENSMUST00000115555	IC 1A*Dnci1a*	1 to 17	AS1	645
Dync1i1.B	NM_010063.3	uc009awn.1, ENSMUST00000115559	IC 1B*Dnci1c*	1 to 17	AS2	628
Dync1i1.D		ENSMUST00000115556	*Dnci1d*	1 to 17	AS3	617
Dync1i1.E		**GU992206** *		1 to 4, 6 to 17	AS1	625
Dync1i1.C		ENSMUST00000115554	IC 1C*Dnci1e*	1 to 4, 6 to 17	AS2	608
Dync1i1.F		**GU992207** *		1 to 4, 6 to 17	AS3	597

The mouse gene nomenclature convention is to name each isoform: genename_GenBank transcript number. We have used the unofficial names Dync1i1.A, Dync1i1.B, etc., here to show their correspondence to the widely used IC 1A, 1B, 1C previously described in rat [22]. However, we also show the correspondence to GenBank mouse cDNAs, UCSC known genes and Ensembl transcripts. As *Dync1i2* has two alternative non-coding first exons, we cannot determine which *Dync1i2* isoforms correspond to the previously described rat isoforms IC2A, 2B, 2C, hence these are in parentheses [22]. Crackower and colleagues noted the presence of some mouse splice isoforms and we also show to their notation from their 1999 publication Dnci1a, c, d, e in italics [34]. We number exons from 1 to 17 for *Dync1i1*, and from 1 to 18 for *Dync1i2* including either exon 1a or exon 1b, and exon 3b.

*first described in this paper.

### Analysis of the splicing pattern of mouse *Dync1i1*

*Dync1i1* is located on chromosome 6 band qA1, spanning over 302,000kb between base pairs 5,675,739 and 5,978,030 (Ensembl, March 2010, transcript ENSMUST00000115555), and exons 1 and exon 17 contain the 5′UTR and 3′UTR respectively; the start methionine is encoded in exon 2 (the exon-intron boundaries of the mouse genomic locus are given in **Table S1**). We found no evidence for alternative first or last exons. A conserved miRNA binding site for miR155 lies in the 3′UTR as determined by TargetScan.

To survey the *Dync1i1* splicing pattern spatially and temporally in mouse, we extracted total RNA from a set of tissues: adult male and female: brain, spinal cord, ovary, testis, spleen, lung, kidney, heart, intestine, muscle, liver; and embryonic E17.5: whole embryos, brain and spinal cord. We synthesised cDNA from oligo dT primers and then amplified PCR products using the primers DIC1_Ex 1 for and DIC1_Ex 17 rev (**Table 2**) that bind within the first and last exons of the longest mouse *Dync1i1* sequence in the UCSC database, uc009awn.1 (RefSeq sequence NM_010063.3).

**Table 2:** Primer pairs used to amplify individual *Dync1i1* splice isoforms (Figure 1).

Primer pair	Isoform detected	Predicted size of amplicon
DIC1_**Ex 1** for and DIC1_**R** rev	Dync1i1.ADync1i1.BDync1i1.DDync1i1.EDync1i1.CDync1i1.F	531 bp480 bp447 bp471 bp420 bp387 bp
DIC1_**Ex 1** for and DIC1_**AS4** rev	Dync1i1.ADync1i1.B	371 bp320 bp
DIC1_**Ex 1** for and DIC1_**5** rev	Dync1i1.ADync1i1.BDync1i1.D	477 bp426 bp393 bp
DIC1_**Ex 1** for and DIC1_**iso14** rev	Dync1i1.EDync1i1.CDync1i1.F	447 bp396 bp363 bp
DIC1_**1.1** for and DIC1_**5** rev	Dync1i1.A	206 bp
DIC1_**1.1** for and DIC1_**iso14** rev	Dync1i1.E	155 bp

**Figure 1:**
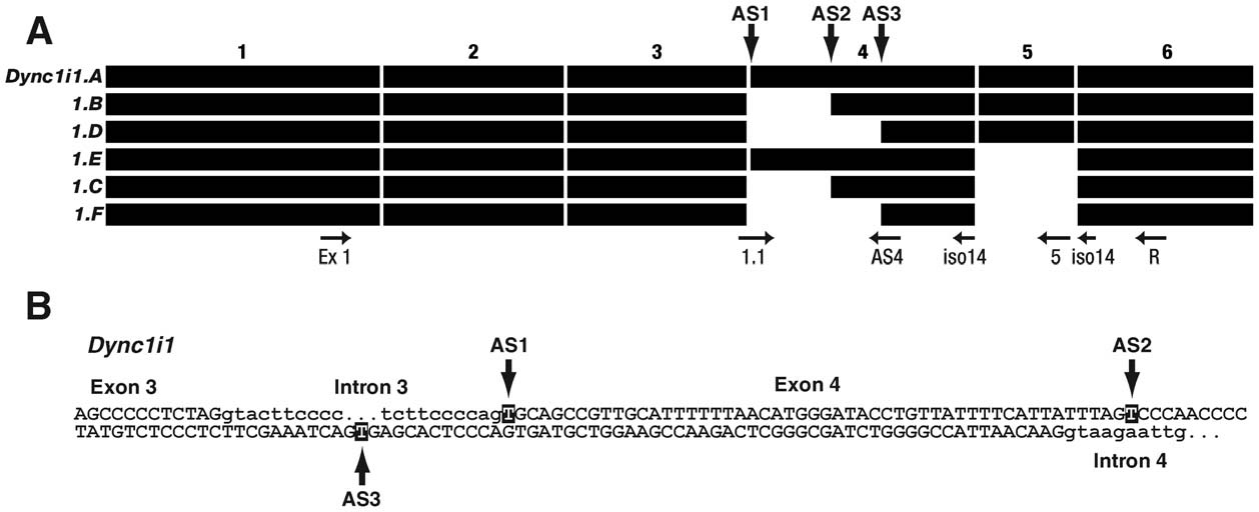
Splicing pattern of mouse dynein intermediate chain 1 gene. **A**. Summary of alternative splicing in *Dync1i1*, showing splice variants *Dync1i1.A* to *Dync1i1.F*, and the first 6 exons (numbered 1, 2, 3, 4, 5, 6); primer binding sites used for isoform specific RT-PCRs are as indicated (**Table 2** **, S2**), and AS1, 2 and 3 are the alternative splice sites in exon 4. Exons are drawn to scale. **B**. Alternative splice sites in *Dync1i1* exon 4. The nucleotide in bold is the first base pair of the exon. Bases in lower case are intronic, bases in upper case are exonic.

We found a complex pattern of amplified fragments in brain, spinal cord, and the embryonic tissues, one homogenously sized amplicon in testis and ovary, and no amplification detectable from spleen, lung, kidney, heart, intestine, muscle or liver. PCR products were sequenced and the amplicon in testis and ovary was found to be a single transcript, here named isoform *Dync1i1.C* (**Figure 1**). The sequences from the adult brain, spinal cord, and E17.5 embryonic tissues were unreadable, presumably due to the presence of multiple splice isoforms. To further investigate, the PCR products from brain and spinal cord were subcloned and sequenced to determine which exons were present in individual transcripts.

From sequencing 62 brain and 14 spinal cord subclones and from a bioinformatics analysis of mouse, human and rat, we found six different transcripts, including two novel isoform (here named *Dync1i1.E* and *Dync1i1.F*) (**Figure 1**). Exon 5 (60 bp) is absent from three of these transcripts (*1.C*, *1.E*, *1.F*). We also found, that exon 4 has three possible alternative splice sites, in agreement with human bioinformatics, while only two splice sites were detected experimentally in rat.

**Figure 2:**
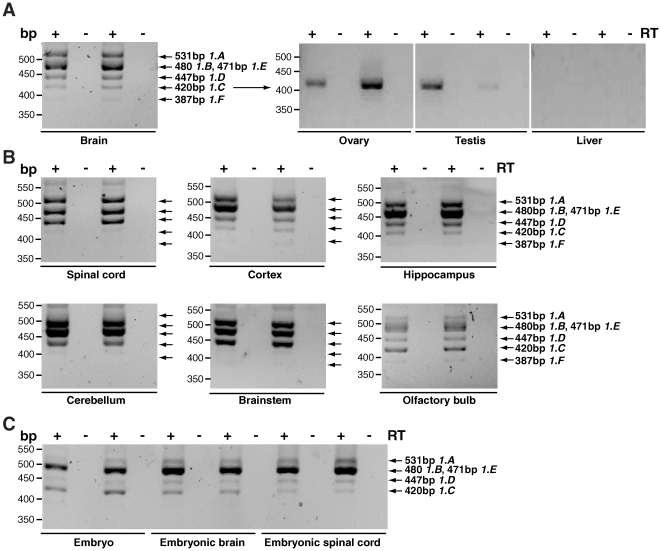
Amplification of different isoforms of *Dync1i1* in mouse tissues using one primer pair. Isoforms of *Dync1i1* were amplified in mouse tissues using primer pair DIC1 Ex1 for and DIC1_R rev which produces six products: *Dync1i1.A* (531 bp), *Dync1i1.B* (480 bp), *Dync1i1.E* (471 bp), *Dync1i1.D* (447 bp), *Dync1i1.C* (420 bp), *Dync1i1.F* (387bp). *Dync1i1.B* (480 bp) and *Dync1i1.E* (471 bp) are not resolved due to similar length of PCR products, and were subsequently individually confirmed as present using isoform specific primer pairs (**Table 2**), for example see amplification of *Dync1i1.E* using isoform specific primer pair DIC1_1.1 for and DIC1_5 rev (**Figure S2**). Note that in some tissues amplification products are faint, and so results were confirmed with other primer pairs as indicated in **Table 2**; for example, see amplification of *Dync1i1.A* in **Figure 3**. In some tissues we also saw a high molecular weight fragment running at approximately 560bp (for example see spinal cord or cerebellum); this may represent another isoform but we were unable to clone or sequence this fragment. **A**. Adult tissues: brain, ovary, testis and liver. In brain 6 amplicons are detected as shown. In ovary and testis we detect isoform *Dync1i1.C* only (420bp). No product is amplified in liver. **B**. Adult neuronal tissues: in spinal cord, cortex, hippocampus, cerebellum, brainstem and olfactory bulb, between four and six amplicons (for example compare brainstem and olfactory bulb) could be detected as shown. **C**. Embryonic tissues: five amplicons are detected as shown. ‘+’ lanes are cDNA, ‘−’ lanes control for genomic DNA contamination and have no reverse transcriptase.

**Figure 3:**
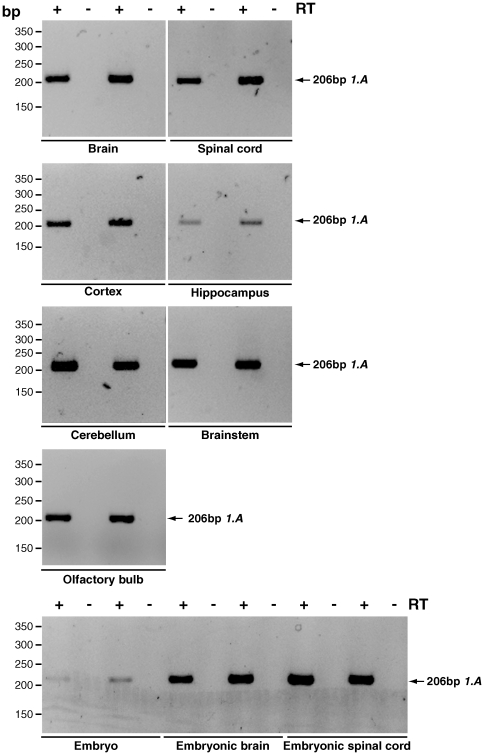
An isoform specific RT-PCR: screening for *Dync1i1.A* in mouse tissues. Primers DIC1_1.1 for and DIC1_5 rev amplify a 206 bp product from isoform *Dync1i1.A*. ‘+’ lanes are cDNA, ‘−’ lanes control for genomic DNA contamination and have no reverse transcriptase. *Dync1i1.A* is found in all brain regions screened, but may not be highly expressed in the E17.5 whole embryo, although this is not a quantitative PCR (data not shown).

To determine which individual isoforms were present in dissected brain regions (cortex, cerebellum, brain stem, hippocampus, olfactory bulb) and other tissues we designed a panel of isoform specific primer pairs (**Table 2**) and surveyed tissues for the presence or absence of the six *Dync1i1* isoforms (for example, **Figures 2** **, S1**). We found our amplification results showed reproducible differences in the patterns of intensity of individual amplicons in different tissues; for example, compare amplification of brainstem and olfactory bulb in **Figure 2B**. Although RT-PCR is not a quantitative method these robust and repeatable amplification patterns probably indicate differences in relative expression of the isoforms in different brain regions. We went on to use nested PCRs to confirm the presence of some individual amplicons which had similar lengths for initial PCRs (for example, **Figures 3**, **S2**). A summary of our findings is presented in **Table 3** and **Figure 1**. In addition we note that for isoform:

**Table 3:** Summary of mouse dynein intermediate gene 1 splice isoform expression in different mouse tissues.

*Dync1i1*						
Tissue	Dync1i1.A	Dync1i1.B	Dync1i1.D	Dync1i1.E	Dync1i1.C	Dync1i1.F
Whole adult brain	+	+	+	+	+	+
Adult spinal cord	+	+	+	+	+	+
Adult cortex	+	+	+	+	+	+
Adult cerebellum	+	+	+	+	+	+
Adult brainstem	+	+	+	+	+	+
Adult hippocampus	+	+	+	+	+	+
Adult olfactory bulb	+	+	+	+	+	+
Whole embryo E17.5	+	+	+	+	+	*
Embryonic brain E17.5	+	+	+	+	+	+
Embryonic spinal cord E17.5	+	+	+	+	+	*
Ovary					+	
Testis					+	
Spleen						
Kidney						
Lung						
Heart						
Intestine						
Muscle						
Liver						

No differences were detected in male and female samples, thus results are not broken down by sex.

*weak amplification detected.

#### *Dync1i1.A*

This isoform was detected originally at low levels in adult brain by RT-PCR and was then cloned and sequenced. This isoform corresponds to the previously described protein, DYNC1I-1A (IC1A) in rat [6], [2], [3]. We confirmed the presence of this isoform in a specific RT-PCR using primers DIC1_1.1 for and DIC1_5 rev to detect a 206 bp amplicon in multiple brain regions and spinal cord, and embryonic tissues but not other tissues in mouse (**Figure 3** **,** **Table 3**). Database searches showed orthologous isoforms in human (uc003uoc.3) and rat (NM_019234.1).

#### *Dync1i1.B*

This isoform was detected in 33/62 subclones from adult mouse brain and 9/14 subclones from adult mouse spinal cord. It is identical to the mouse cDNA uc009awn.1 and corresponds to the previously described protein, IC1B in rat [21], [23]. Dync1i1.B is expressed in the nervous system and embryonic tissues only (**Table 3**). Database searches found an orthologous full-length cDNA in human (uc003uod.3), but only ESTs in rat (CB703089, CB747457).

#### *Dync1i1.D*

This isoform was detected in 16/62 subclones from adult mouse brain and 5/14 subclones from adult mouse spinal cord, and was amplified in these tissues and embryonic tissues using two different sets of primer pairs (DIC1_Ex 1for and DIC1_R rev, DIC1_Ex 1 for and DIC1_5 rev, **Table 2**) but did not amplify in other tissues. This isoform has been found as a human EST (DA392898) but is not present in the current rat databases.

#### *Dync1i1.E*

This novel isoform has not been characterized previously. It was not detected during the initial cloning analysis nor was it present amongst the UCSC mouse sequences (mm9). However, by database searching we found a human cDNA (uc003uoe.3) and a rat EST (CB712052) in which AS1 in exon 4 is present and exon 5 is removed. Therefore we designed PCR primers (DIC1_1.1 for and DIC1_iso14 rev) to detect only this isoform in mouse. In all brain tissues examined plus spinal cord and embryonic tissues, the expected 155 bp product was amplified (for example see **Figure S2**). This isoform was not found outside of brain or spinal cord, or embryonic tissues. The GenBank accession number obtained for this sequence is GU992206 (**Table 1**).

#### *Dync1i1.C*

This isoform was detected in 12/62 subclones from adult mouse brain and unlike the other *Dync1i1* transcripts it was found in ovary and testis, but not other tissues (in amplification with the primer pairs DIC1_Ex 1 for and DIC1_R rev or DIC1_Ex 1 for and DIC1_iso14 rev) (**Figure S1**). It corresponds to the previously described protein IC 1C in rat. This isoform has a human full-length orthologue, uc003uob.2, and is homologous to two rat ESTs CB616735, CB713291.

#### *Dync1i1.F*

This novel isoform was detected in 1/62 subclones from brain, and amplified at low levels throughout the nervous system and embryonic tissues, with primer pairs DIC1_Ex 1 for and DIC1_R rev, and DIC1_Ex 1 for and DIC1_iso14 rev, but it does not amplify in other tissues. We did not find orthologous human or rat sequences in the databases. This is a low abundance isoform in the tissues assayed and it remains unknown if it is a functional transcript. The GenBank accession number obtained for this sequence is GU992207 (**Table 1**).

### Analysis of the splicing pattern of mouse *Dync1i2*

*Dync1i2* maps to chromosome 2, band C2, spanning over 51,000kb between base pairs 71,049,798 and 71,101,360 (Ensembl, March 2010, transcript ENSMUST00000112140). The start methionine lies in exon 2. We did not detect any evidence for microRNA binding sites in the 3′UTR of the gene (the exon-intron boundaries of the mouse genomic locus are given in **Table S1**).

Bioinformatics analysis of the *Dync1i2* gene in mouse, human and rat showed that in all three species this gene produces two sets of transcripts containing different first exons, indicating the presence of two independent promoters; this is corroborated by analysis with the DBTSS track of the UCSC Genome Browser which distinguishes two active promoters, adjacent to two non-coding exons in the three species. We named the two alternative exons 1a and 1b. Exon 1a is upstream of Exon1b and they are separated by ∼125bp of genomic DNA with no overlap between the exons. We found no transcript that contained both exons. We note that the start methionine remains the same in all *Dync1i2* transcripts as this lies in exon 2.

To survey *Dync1i2* splicing we designed two RT-PCRs based on sequences in the UCSC Genome Browser (exon 1a: uc008kah.1, uc008kai.1; exon 1b: uc008kak.1, uc008kaj.1). We amplified sequence using forward primers from either exon 1a (DIC2_Ex1a for) or exon 1b (DIC2_Ex1b for) and one reverse primer lying in the last exon of the gene (DIC2_Ex18 rev) (Primer sequences used to determine the splicing patterns of *Dync1i1* and *Dync1i2* are given in **Table S2**). Amplified products of *Dync1i2* from both primer pairs were purified and sequenced.

**Figure 4:**
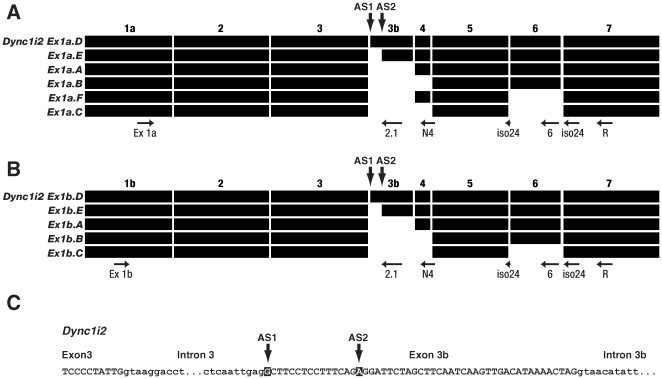
Splicing pattern of mouse dynein intermediate chain 2 gene. **A, B**. Summary of alternative splicing in *Dync1i2* with exon 1a or 1b respectively and the first seven exons (numbered 1a or 1b, 2, 3, 3b, 4, 5, 6, 7). Primer binding sites used for isoform specific RT-PCRs are as indicated (**Table 4** **, S2**). AS refers to alternative splice sites in exon 3b. Note that we could not detect the presence of the putative *Dync1i2(1b).F* in any tissue surveyed. Exons are drawn to scale. **C**. Alternative splice sites in *Dync1i2* exon 3b. The nucleotide in bold is the first base pair of the exon. Bases in lower case are intronic, bases in upper case are exonic.

In all non-neuronal and non-embryonic tissues, we found that primers specific for either exon 1a or exon1b yielded a single isoform designated 2C in this paper i.e. *Dync1i2(1a).C* or *Dync1i2(1b).C* (**Figure 4**). However, the amplification patterns in the nervous system and embryonic tissues were complex and we were unable to sequence the mix of PCR products from these tissues. To determine which isoforms of *Dync1i2* were expressed in these tissues, the RT-PCR products were subcloned and sequenced; subsequent analysis, revealed a total of 11 different isoforms each having either exon 1a or exon 1b at the start of the transcript (**Table 1**, **Figure 4**). This Includes two novel isoforms (*Dync1i2(1a).D* and *Dync1i2(1b).D*, **Figure 4**) that were detected during RT-PCR analysis.

**Figure 5:**
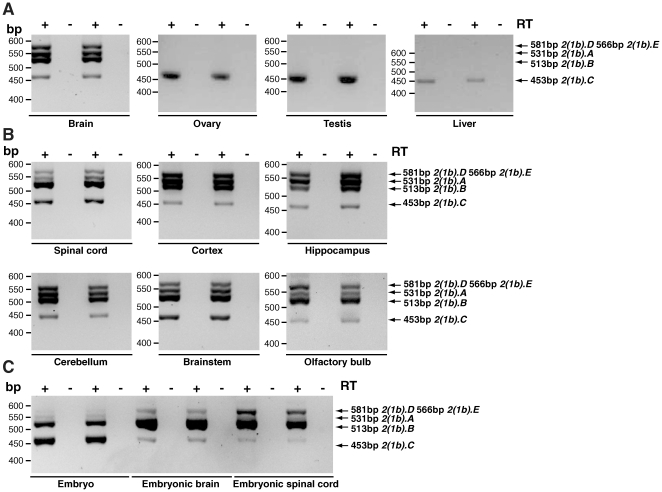
Amplification of different isoforms of *Dync1i2* Exon 1b in mouse tissues using one primer pair. Isoforms of *Dync1i2* Exon 1b were amplified in mouse tissues using primer pair DIC2_Ex 1b for and DIC2_R rev which produce five products: *Dync1i2.D* (581 bp), *Dync1i2.E* (566 bp), *Dync1i2.A* (531 bp), *Dync1i2.B* (513 bp) and *Dync1i2.C* (453 bp). *Dync1i2.D* (581 bp) is not resolved from *Dync1i2.E* (566 bp) due to the similar length of PCR products; therefore we undertook nested PCRs with primers DIC2_Ex 1b for and DIC2_2.1 rev to determine the transcription pattern of these two isoforms (data not shown). We did not detect 2*(1b).F* in this assay. **A**. Adult tissues: brain, ovary, testis and liver. In brain five amplicons are detected as shown (and confirmed by nested PCRs). In ovary, testis and liver we detect isoform *Dync1i2.C* only. **B**. Adult neuronal tissues: In spinal cord, cortex, hippocampus, cerebellum, brainstem and olfactory bulb five amplicons are detected as shown. **C**. Embryonic tissues: Three or five amplicons are detected as shown.

Based on the information from individual subclones derived from both exons 1a and 1b, we then designed isoform specific primer pairs to survey all mouse tissues previously described; we used nested PCRs to confirm the presence of some individual amplicons whch had similar lengths in our intial PCRs (**Table 4** **,** **Figure 5** **,** **6**).

**Figure 6:**
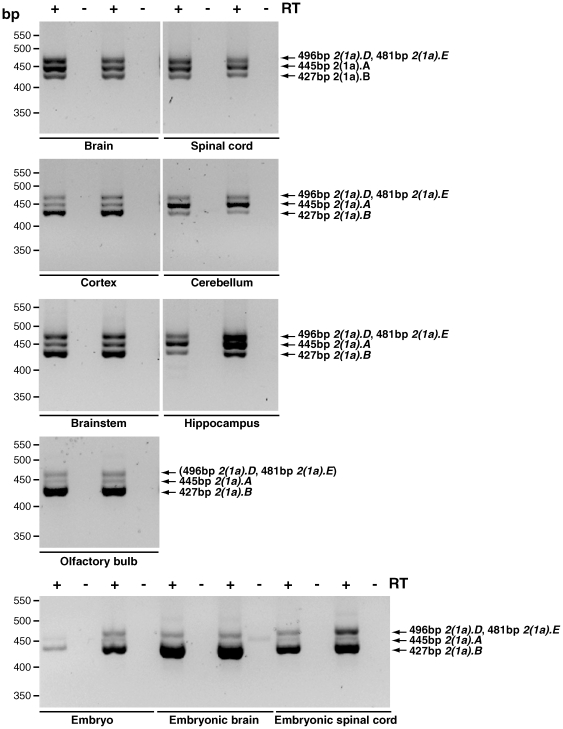
Examples of Dync1i2 Exon 1a isoform specific PCR. Four isoforms of *Dync1i2* Exon1a are amplified in the mouse nervous system and E17.5 embryonic nervous tissues using primer pair DIC2_Ex1a for and DIC2_6 rev. Amplicons are as shown and in addition while *Dync1i2(1a).D* was not resolved in this amplification it was amplified as shown in Table 5 using primers DIC2_Ex 1a for and DIC2_2.1 rev (data not shown). We were unable to resolve, even by nested PCR, which of isoforms *Dync1i2(1a).D* and *Dync1i2(1a).E* were present in olfactory bulb. ‘+’ lanes are cDNA, ‘−’ lanes control for genomic DNA contamination and have no reverse transcriptase.

**Table 4:** Primer pairs used to amplify individual *Dync1i2* splice isoforms (Figure 4).

*Dync1i2* exon1a splice isoforms		
Primer pair	Isoform detected	Predicted bands
DIC2_**Ex 1a** for and DIC2_**R** rev	Dync1i2(1a).DDync1i2(1a).EDync1i2(1a).ADync1i2(1a).B(Dync1i2(1a).F)Dync1i2(1a).C	556 bp541 bp505 bp487 bp445 bp427 bp
DIC2_**Ex 1a** for and DIC2_**2.1** rev	Dync1i2(1a).DDync1i2(1a).E	315 bp300 bp
DIC2_**Ex 1a** for and DIC2_**N4** rev	Dync1i2(1a).DDync1i2(1a).EDync1i2(1a).A(Dync1i2(1a).F)	352 bp337 bp301 bp301 bp
DIC2_**Ex 1a** for and DIC2_**6** rev	Dync1i2(1a).DDync1i2(1a).EDync1i2(1a).ADync1i2(1a).B	496 bp481 bp445 bp427 bp
DIC2_**Ex 1a** for and DIC2_**iso24** rev	(Dync1i2(1a).F)Dync1i2(1a).C	405 bp387 bp

*Dync1i2* exon1b splice isoforms		
DIC2_**Ex 1b** for and DIC2_**R** rev	Dync1i2(1b).DDync1i2(1b).EDync1i2(1b).ADync1i2(1b).B(Dync1i2(1b).F)Dync1i2(1b).C	581 bp566 bp531 bp513 bp(471 bp)453 bp
DIC2_**Ex 1b** for and DIC2_**2.1** rev	Dync1i2(1b).DDync1i2(1b).E	340 bp325 bp
DIC2_**Ex 1b** for and DIC2_**N4** rev	Dync1i2(1b).DDync1i2(1b).EDync1i2(1b).A(Dync1i2(1b).F)	378 bp363 bp(327 bp)
DIC2_**Ex 1b** for and DIC2_**6** rev	Dync1i2(1b).DDync1i2(1b).EDync1i2(1b).ADync1i2(1b).B	522 bp507 bp471 bp453 bp
DIC2_**Ex 1b** for and DIC2_**iso24** rev	(Dync1i2(1b).F)Dync1i2(1b).C	(431 bp)413 bp

Note that we have been unable to detect the *Dync1i2* exon 1b.F.

We also detected a novel exon of 51bp, here designated exon 3b, which lies downstream of exon 3; this has two alternative splice sites (**Figure 4**). Exon 3b does not align to human genomic sequence from either dynein intermediate chain gene but does appear to have an equivalent sequence in rat genomic DNA in *Dync1i2*. In mouse, when we align exon 3b with *Dync1i1* genomic sequence, aligning either DNA or the translations, we are unable to detect a likely equivalent exon, thus this exon 3b appears specific for *Dync1i2* in mouse and rat (and see Discussion).

### Summary of mouse *Dync1i2* alternative splicing

A summary of our findings is presented in **Table 5**, **Figure 4**. We found *Dync1i2* has 11 isoforms: six including exon 1a, five including exon 1b; isoforms with the same exon content other than exon 1a and 1b have similar but not identical expression patterns (**Table 5**). In addition we note that for isoform:

**Table 5:** Summary of mouse dynein intermediate gene 2 splice isoform expression in different mouse tissues.

*Dync1i2* Exon 1a						
Tissue	Dync1i2.D	Dync1i2.E	Dync1i2.A	Dync1i2.B	Dync1i2.C	Dync1i2.F
Whole adult brain		+	+	+	+	+
Adult spinal cord	+	+	+	+	+	
Adult cortex	+	+	+	+	+	
Adult cerebellum		+	+	+	+	
Adult brainstem	+	+	+	+	+	
Adult hippocampus		+	+	+	+	
Adult olfactory bulb			+	+	+	
Whole embryo E17.5			+	+	+	
Embryonic brain E17.5	+	+	+	+	+	
Embryonic spinal cord E17.5	+	+	+	+	+	
Ovary					+	
Testis					+	
Spleen					+	
Kidney					+	
Lung					+	
Heart					+	
Intestine					+	
Muscle					+	
Liver					+	

*Dync1i2* Exon 1b					
Tissue	Dync1i2.D	Dync1i2.E	Dync1i2.A	Dync1i2.B	Dync1i2.C
Whole adult brain	+	+	+	+	+
Adult spinal cord	+	+	+	+	+
Adult cortex		+	+	+	+
Adult cerebellum	+	+	+	+	+
Adult brainstem	+	+	+	+	+
Adult hippocampus	+	+	+	+	+
Adult olfactory bulb	+	+	+	+	+
Whole embryo E17.5		+	+	+	+
Embryonic brain E17.5		+	+	+	+
Embryonic spinal cord E17.5		+	+	+	+
Ovary					+
Testis					+
Spleen					+
Kidney					+
Lung					+
Heart					+
Intestine					+
Muscle					+
Liver					+

No differences were detected in male and female samples, thus results are not broken down by sex.

#### *Dync1i2(1b).D*

This novel isoform was identified as an unexpected larger 340 bp product that was produced in addition to the expected *Dync1i2(1b).E* (325 bp) amplicon when some but not all nervous system tissues were amplified with the primers DIC2_Ex 1b for and DIC2_2.1 rev. The fragment was sequenced and found to contain the new exon, 3b, which is flanked by consensus splice sites and has two alternative splice acceptor sites. We did not find an alignment of exon 3b in the human genomic dynein intermediate chain genes. However, in rat, while no equivalent transcripts are in the databases, we did detected a 48 bp almost perfect alignment to rat genomic sequence in the expected position within rat intron 3, except that an internal 3bp (GAG, encoding glutamic acid), is absent. The complete 48 bp can be transcribed into a 16 amino acid peptide that maintains the open reading frame of the protein and thus this is likely to be rat exon 3b. *Dync1i2.D* is the longest *Dync1i2(1b)* transcript we can detect, being 2,478 bp in length. This isoform does not so far have orthologs in human or rat. GenBank accession number obtained for this sequence is GU992211 (**Table 1**).

#### *Dync1i2(1a).D*

This novel isoform was amplified using primers spanning Exon 1a and exon DIC2_Ex 1a for and DIC2_2.1 rev to determine if an equivalent to *Dync1i2 Exon 1b.D* was expressed. The isoform was found in some but not all nervous system tissues, and in embryonic nervous system but not whole embryo. It is the longest *Dync1i2(1a)* transcript we can detect, being 2,493 bp in length. This isoform does not so far have orthologs in human or rat. The GenBank accession number obtained for this sequence is GU992208 (**Table 1**).

#### *Dync1i2(1a).E*

This novel isoform was detected based on the sequence of *Dync1i2(1b).E*, in all nervous system tissues except for olfactory bulb, and embryonic tissues, using PCR with primers DIC2_Ex 1a for and DIC2_6 rev and then nested PCR with DIC2_Ex 1a for and DIC2_2.1 rev and sequencing of the amplified 300 bp product. This isoform does not so far have orthologs in human or rat and we note that *Dync1i2(1b).E* is a low abundance transcript. The GenBank accession number obtained for this sequence is GU992209 (**Table 1**).

#### *Dync1i2(1b).E*

This novel isoform was found as one clone out of 64 in brain; it has not been detected so far in human transcripts and it may be a low abundance transcript that is specific to the mouse/rat evolutionary branch. The GenBank accession number obtained for this sequence is GU992212 (**Table 1**).

#### *Dync1i2(1a).A*

This isoform was detected in 36/75 brain subclones and 8/82 spinal cord subclones. It corresponds to mouse cDNA ENSMUST00000112140 and has an homologous full-length human cDNA sequence uc002uha.1 and is also described by a rat EST CB784025. It is detected by RT-PCR with the primer pairs DIC2_Ex 1a for and DIC2_R rev, DIC2_Ex 1a for and DIC2_N4 rev, and DIC2_Ex 1a for and DIC2_6 rev (**Table 4**).

#### *Dync1i2(1b).A*

This isoform was detected in 35/64 brain subclones and 17/72 spinal cord subclones; it corresponds to the human cDNA uc002uhe.1 and rat cDNAs RefSeq NM_053880, and GenBank mRNA U39044. It is amplified by the primer pairs DIC2_Ex 1b for and DIC2_R rev, DIC2_Ex 1b for and DIC2_N4 rev and DIC2_Ex 1b for and DIC2_6 rev.

#### *Dync1i2(1a).B*

This isoform was detected in 16/75 brain subclones and 17/82 spinal cord subclones. It is shorter than isoform *Dync1i2(1a).A* by 18 bp as a result of removing of exon 4. This isoform is described by a number of human ESTs (BP228682, BP229314, DA386957, DA488835, DA492636, DA497525, DA694727, DA696722, DA749926, DA769782, DA776852, DB003699, DB038590, DA493266, DA487646, DB173651) and rat EST CF977964. It is detected by primer pairs DIC2_Ex 1a for and DIC2_R rev and DIC2_Ex 1a for and DIC2_6 rev.

#### *Dync1i2(1b).B*

This isoform was detected in 15/64 brain subclones and 23/72 spinal cord subclones. It is shorter than isoform *Dync1i2(1b).A* by 18 bp as a result of removing of exon 4 and it corresponds to human full-length cDNA uc002uhd.1 and rat GenBank mRNA U39045, ESTs CB711834, CK470011, CV107841 and in mouse is detected by primer pairs DIC2_Ex 1b for and DIC2_R rev, and DIC2_Ex 1b for and DIC2_6 rev.

#### *Dync1i2(1a).F*

This novel isoform was detected in 1/75 brain subclones, in mouse only – no corresponding human or rat expressed sequences have been found so far, thus this may be a low abundance isoform or an aberrant splice product without functional significance. This isoform should be detected in mouse with primer pairs DIC2_Ex 1a for and DIC2_R rev, DIC2_Ex 1a for and DIC2_N4 rev and DIC2_Ex 1a for and DIC2_iso24 rev, however we were not able to detect it in any tissue by PCR. The GenBank accession number obtained for this sequence is GU992210.

#### Putative *Dync1i2(1b).F*

We are unable to determine if this exists. We tried several times in each tissue to detect the presence of isoform *Dync1i2(1b).F* but were consistently unable to do so. We cannot detect it by RT-PCR or in any of our subclones, nor is it present in the current human or rat transcript databases. This may show this isoform is not expressed. However, we note that the *Dync1i2* Exon 1a.F equivalent is also detected at low abundance.

#### *Dync1i2(1a).C*

This isoform was detected in 19/75 brain subclones and 53/82 spinal cord subclones and all other tissues, it corresponds to human cDNA uc002uhb.1 and rat ESTs CK365527, EV774963, FM056664. It is detected in mouse with primer pairs DIC2_Ex 1a for and DIC2_R rev, and DIC2_Ex 1a for and DIC2_iso24 rev.

#### *Dync1i2(1b).C*

This isoform was detected in 13/64 brain subclones and 36/72 spinal cord subclones, and all other tissues, it corresponds to human uc002uhf.1 and rat GenBank mRNA U39046 and ESTs CD568113, CK355960, CK367553, CK471588, CV108862, EV763033, EV774374. It is detected using mouse primers DIC2_Ex 1b for and DIC2_R rev, and DIC2_Ex 1b for and DIC2_iso24 rev.

## Discussion

We undertook an extensive survey of the expression of mRNAs of the cytoplasmic dynein intermediate chain genes *Dync1i1* and *Dync1i2* in a range of mouse tissues. We utilized RT-PCR on mouse transcripts to show the presence of new intermediate chain isoforms in the mouse including those derived from novel exons and non-coding exons. We found the greatest complexity to be present in the adult nervous system. Bioinformatic analyses corroborate the existence of these complex splicing patterns and new isoforms and confirm the similarities between mouse, human and rat. These analyses were performed at the tissue, not cellular, level and other less abundant isoforms may remain to be identified, particularly those expressed earlier in development than E17.5. For example, we detected at a low level from mouse, cDNAs of *Dync1i2* isoforms that are deleted for a single glutamine residue in exon 18 (data not shown, Ensembl transcripts ENSMUST00000112136, ENSMUST00000112139), and we find the presence of two other possible new isoforms present as full-length cDNAs in human (uc010zdt.1 (exon 1a), uc010zds.1 (exon 1b), hg19, GRCh37), however, these do not appear to be expressed in mouse or rat (A.Kuta, unpublished). These transcripts contain an exon 5 which is 54 bp longer than the exon 5 shown here because of the use of an upstream alternative 3′ splice site; and at the same time exons 4 and 6 are excluded, which results in a protein of 630 amino acids in length.

Prior to this work, the distribution and expression of intermediate chain splice isoforms had been best studied in the rat [21], [22], [23], [24]. However, dynein intermediate chain splice isoforms are not restricted to mammals or vertebrates. For example, complex splicing patterns are found in the homologous intermediate chain gene in *Drosophila*, *Cdic* [25] and these patterns arise from alternative splicing of three small exons located between exons 4 and 5 of this gene. This strongly suggests that alternative splicing of the N-terminal region of the intermediate chains is essential for some aspect of eukaryotic dynein motor protein function in cells.

### Alternative 5′ non coding exons in *Dync1i2*

**Figure 7:**
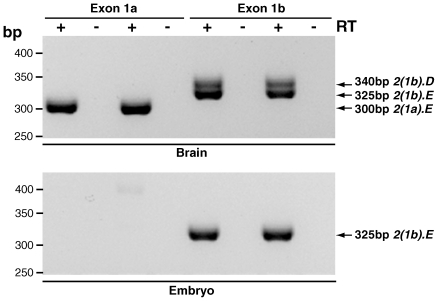
Differential expression of *Dync1i2* with exon 1a and exon1b containing isoforms. Brain cDNA was amplified by nested RT-PCR with exon 1a primers DIC2_Ex 1a for and DIC2_2.1 rev which amplify *2(1a).D* (315bp) and *2(1a).E* (300bp), and with exon 1b primers DIC2_Ex 1b for and DIC2_2.1 rev which amplify *2(1b).D* (340bp) and *2(1b).E* (325bp). We detect *Dync1i2.E* containing exons 1a or 1b; we detect *Dync1i2.D* containing exon 1b only and not exon 1a, showing differential expression of this isoform depending on which first non-coding exon is spliced in the adult brain. E17.5 whole embryo cDNA was amplified the same primer pairs (DIC2_Ex1a and DIC2_2.1 rev, and DIC2_Ex 1b and DIC2_2.1rev). We detect no *Dync1i2.D* isoforms. However, we detect *2(1b).E* but not *2(1a).E*, showing differential expression of *Dync1i2.E* depending on which first non-coding exon is spliced in.

The presence of two alternative 5′ non-coding first exons in *Dync1i2* is intriguing and suggests another complex layer of regulation. We detected differences in the relative expression levels of *Dync1i2* isoforms depending on whether exon 1a or 1b was spliced into the transcript (**Table 5**); for example see the expression of *Dync1i2(1a).E* and *2(1b).E* in adult brain and whole embryo (**Figure 7**). As the human and mouse genomes are analyzed an increasing number of genes are found to have alternative non-coding first exons [26]. Recent studies in human have found approximately 50% of genes have at least one alternative promoter and such genes tend to have significantly longer genomic structures including 5′ and 3′ UTRs, intron and exon lengths and numbers of exons compared to genes with single promoters ([27] and references therein). Interestingly *Dync1i1* is a significantly longer gene than *Dync1i2*, but so far we can find no evidence for it having an alternative non-coding 5′ exon. The use of alternative promoters is thought to be associated with cell-specific expression; for example the human CYP19 aromatase gene has at least 10 alternative non-coding first exons [28] which appear to control mRNA stability and gene expression [29]. Similarly, the use of three (in mouse and human) or two (in rat) alternative promoters resulting in the expression of alternative non-coding first exons has been shown for GATA4 gene [30].

### Significance of the different intermediate chain isoforms

We have shown that different tissues express different intermediate chain isoforms. All of the *Dync1i1* isoforms were found only in nervous tissue, with the exception of *Dync1i1.C*, which was also found in ovary and testis. This result is consistent with previous studies in rat that found that all the IC1 isoforms are expressed exclusively in the nervous system, except that one was found in testis, and ovary was not examined, [21], [22], [23]. We further found that most mouse tissues express only the IC2C isoforms; this is different from rat, where most tissues express both IC2C and IC2B [21], [22], [23].

While our RT-PCR results are not quantitative, there are clearly reproducible differences between tissues, and our studies further indicate the isoforms also expressed at different levels in different tissues. Further, preliminary data using fluorescent primers and capillary gel electrophoresis to determine the relative amounts of each amplicon in the total amplified pool, showed tissue specific differences in expression (Kuta, A. unpublished). The patterns we see consistently show that when RT-PCR results for *Dync1i1* and *Dync1i2* isoforms are compared between different brain regions the relative proportions of the different isoforms are different (for example, see **Figures 2** **,** **5**).

Most of the splice isoform complexity we observed for both intermediate chains is within the nervous system tissue. This is consistent with the previously published data from rat where all six identified intermediate chain isoform were expressed in the adult brain. In rat, the analysis of the expression of the isoforms was extended to the cellular level and these data demonstrated that the basis for the complexity was in the neurons. Cultured embryonic cortical and hippocampal neurons expressed the IC1B, 1C, 2B, and 2C isoforms. All other cultured cells, including glia, expressed only the IC2C isoform, except that IC2B was found in neuroblastomas [22], [23], [31].

The cytoplasmic dynein complex is involved in many processes including protein and RNA transport, organelle trafficking, as well as mitosis, endosome sorting, and the cellular stress response and response to hypoxia. The neuronal intermediate chain complexity may reflect the dependence of axonal and cellular survival on the efficient transport of trophic and other factors from the synapse to the cell body (often over 1m in length) [32], [11], [33]. It also may reflect the complexity of the different types of cargoes that have to be actively transported within neurons, both in the adult and in the developing embryo. Axonal transport must be a highly regulated and ordered process to transport such disparate organelles and cargo. While the precise role of the intermediate chains in dynein regulation in these processes is not completely understood, it is known that dynein complexes with specific intermediate chains are responsible for the transport of individual organelles. For example dynein complexes with IC1B, are necessary for the transport of Trk signalling endosomes in rat hippocampal axons [10], [21], [22], [23]. At the minimum, the intermediate chain isoforms produce functionally distinct classes of dynein complexes that may be separately regulated to carry different specific cargoes.

Consistent with a role for the intermediate chains in neuron specific function, we clearly observed differences in the expression of intermediate chain isoforms when the patterns from late embryonic and adult brain are compared. In rat only the generic IC2C was found at E13, but with increasing embryonic age, first IC2B and then IC1B and 1C were found. While the IC2A and 1A isoforms were found only in the adult (P20 and older) [23]. Using probes for specific for common regions of IC1 and IC2, Crackower and colleagues investigated dynein intermediate chain expression in mouse embryos at different stages of development using whole mount in situ hybridization. They found that at mouse E10.5–13.5 *Dync1i1* was restricted to the developing cortex and the peripheral nervous system (dorsal root ganglia and sympathetic ganglia), while *Dync1i2* was more widely expressed within the nervous system [34]. In addition *Dync1i2* was found to be highly expressed a non neuronal tissue, the mouse developing limb bud [34], and may play a role in establishing cell polarity by orientating intracellular components correctly [35], [3].

### Relationship between exons and protein domains for dynein intermediate chains

**Figure 8:**
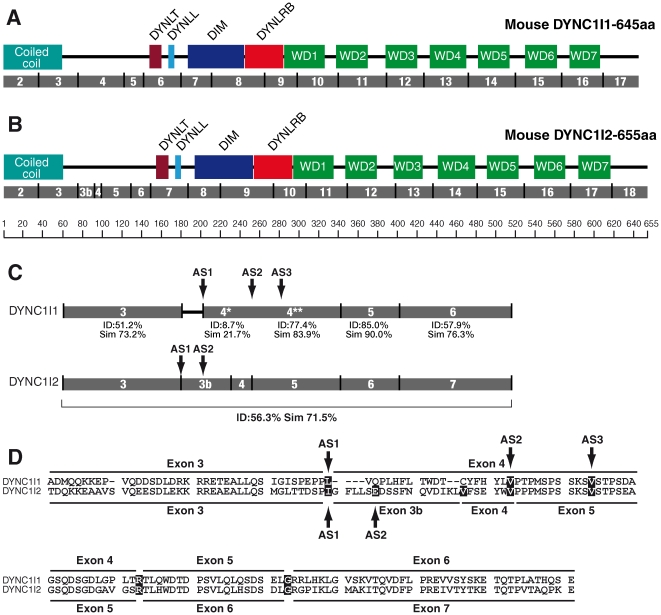
Exon – protein domain relationship for dynein intermediate chain genes. Protein domains marked as blocks, numbered exons marked in grey. The coiled coil region where protein interactions occur is shown as is DYNLT – binding region of Tctex dynein light chains, DYNLL – binding region of LC8 dynein light chains, DIM – intermediate chain dimerisation domain, DYNLRB – binding domain of Roadblock dynein light chains; seven WD40 repeats marked in green. The domain boundaries are as shown in **Table S3** [19], [8], [20], [39], [40], [24], [41]. **A**. Mouse DYNC1I1 isoform 1.A (645 amino acids). **B**. Mouse DYNC1I2 isoform 2.D (655 amino acids). Note that isoform 2.D includes exons 1 to 3 and new exon 3b; exon 4 to 18. **C**. Schematic alignment of DYNC1I1 and DYNC1I2 protein sequences coded by exons 3 to 6 of *Dync1i1* and exons 3 to 7 of *Dync1i2* genes; alternative splice sites in *Dync1i1* exon 4 (AS1, AS2, AS3) and *Dync1i2* exon 3b (AS1, AS2) are marked. The percent of identity (ID) and similarity (Sim) between segments of protein sequences coded by specific exons were compared using AliSubSimP. Protein sequences of DYNC1I1 and DYNC1I2 encoded by exons 3 are 51.2% identical and 73.2% similar; protein sequence of DYNC1I1 exon 4 was compared as two segments marked with asterix: 4* from AS1 to AS2 and 4** from AS2 to the end of exon 4. There is only 8.7% identity and 21.7% similarity between *Dync1i1* exon 4* and DYNC1I2 exons 3b and 4 while DYNC1I1 exon 4** and DYNC1I2 exon 5 show 77.4% identity and 83.9% similarity. Furthermore, DYNC1I1 exon 5 is 85.0% identical and 90.0% similar to DYNC1I2 exon 6. Protein sequences of DYNC1I1 encoded by exon 6 and DYNC1I2 encoded by exon 7 are 57.9% identical and 76.3% similar. The whole fragments of the intermediate chains presented show 56.3% identity and 71.5% similarity. **D**. Alignment of DYNC1I1 and DYNC1I2 protein sequences encoded by exons 3 to 6 of *Dync1i1* and exons 3 to 7 of *Dync1i2* genes; alternative splice sites in *Dync1i1* exon 4 (AS1, AS2, AS3) and in *Dync1i2* exon 3b (AS1, AS2) marked, amino acids shaded overlap splice sites.

The intermediate chains have a scaffold-like role in the dynein complex. They dimerize and bind to the dynein heavy chain, the three light chain dimers, and the putative cargo adaptor, dynactin. The intermediate chains and the light chains, with the light intermediate chains, form the cargo binding domain of the cytoplasmic dynein complex [3], [36], [37]. The intermediate chain N-terminal region forms a coiled-coil which interacts with p150 subunit of dynactin [24]. At the C-terminal lies seven WD40 repeats which are thought to form a β propeller structure responsible for interaction with the dynein heavy chain [20], [38]. Proximal to the WD repeats are the binding sites for the dynein light chains and the dimerisation domain [19], [8], [39], [40], [41]. As can be seen in **Figure 8**, (the position of domains in the longest DYNC1I1 and DYNC1I2 isoforms are shown in **Table S3**) the exon structure for these major functional domains is similar between the IC1 and IC2 proteins. The alternative splice isoforms of IC1 and IC2 affect the region of the protein downstream of the N-terminal coiled coli region and upstream of the region binding to DYNLT and DYNLL light chains, between exons 3b/4 and exon 5. This region of the N-terminus has been implicated in binding of the p150 subunit of dynactin, and it is rich in serines and threonines, leading to suggestions that one function of the alternative splicing is to generate novel phosphorylation sites that are important for specific dynein regulation, for example in modifying binding to the p150 subunit of dynactin, or other putative cargo adaptors [24], [42]. Our observation that there is only 8.7% identity between IC1 exon 4* (the fragment of exon 4 spanning between the start of this exon and the AS2 site) and IC2 exons 3B and 4 compared to greater than 51% identity for other exons (**Figure 8D**) shows that this region is one of significant variability between the intermediate chains and it is consistent with the hypothesis that this region of the intermediate chains may have an important role in dynein regulation.

### Conclusion

The mechanisms of how dynein intermediate chains contribute to the specificity of dynein function and its regulation are poorly understood. To better understand intermediate chain variation, we have characterized in detail the alternative splicing of the two cytoplasmic dynein intermediate chains genes in the mouse. We have identified new splicing patterns, a novel exon, and the presence of two distinct first exons in *Dync1i2*. Mouse genetic studies have shown that neurons are particularly dependent on the efficient function of cytoplasmic dynein. Consistent with this we have further shown that in the mouse the expression pattern of intermediate chain alternative splicing is richest (or more complex) in neural tissue. This work has important implications for the regulation of cytoplasmic dynein in neurons and will allow additional experiments to further probe the mechanisms of neuronal dynein regulation.

## Materials and Methods

### Mouse tissues

All tissues were collected from C57BL/6J inbred mice, supplied by Harlan Laboratories. Mice were housed in controlled conditions in accordance with guidance issued by the Medical Research Council in *Responsibility in the Use of Animals for Medical Research* (1993) and all experiments were carried out under Licence from the UK Home Office and with full ethical approval from the MRC Prion Unit Local Ethical Review Panel.

For RNA extraction tissues were collected from 2 male and 2 female mice aged 6–8 weeks (brain, spinal cord, ovary, testis, spleen, lung, kidney, heart, intestine, muscle, liver). Brain was also collected from 2 male and 2 female mice aged 6–8 weeks and was dissected into cortex, hippocampus, cerebellum, brainstem, olfactory bulb. Immediately after dissection tissues were placed in RNAse free 1.5ml tubes (Biopur, Eppendorf) and immersed in RNAlater reagent (Qiagen) to minimize RNA degradation. Tissues were stored at 4°C overnight and transferred to −80°C for long term storage.

For RNA extraction from embryonic tissues, timed matings were set up by placing a male overnight with females and removing the male the following day. Embryos were collected from terminally anaesthetized pregnant females at E17, and 2 whole embryos and 2 embryonic brain and spinal cords were collected for this study as above.

### RNA preparation

Tissues were weighed and RNA Mini Kits (Qiagen) were used to extract total RNA from mouse tissues according to manufacturer's instructions, in RNAse-free conditions.

### Preparation of muscle and liver RNA

We found muscle and liver gave poor quality preparations, possibly owing to either the fibrous nature of these tissues or high lipid content in liver, therefore we modified the RNA preparation for these tissues by homogenizing them in TRIreagent (Sigma) at 1ml per 30 mg tissue. Homogenates were centrifuged at 4°C for 30 min at 5,000×g, and supernatant was transferred to a fresh tube for treatment with RNAse free Mussel Glygogen (Sigma) for 5 min at a final concentration of 250 ug/ml, room temperature, after which 0.1 ml bromo-chloro-propane (BCP, Sigma) per 1 ml of TRIreagent was added, the mixture was shaken vigorously and incubated for 2–3 min at room temperature. After centrifugation at 4°C for 30 min at 5,000×g the lysate separated into into 3 phases. The upper aqueous phase was carefully transferred to a fresh tube and 0.3 ml isopropanol and 0.2 ml high salt solution (0.8M sodium citrate, 1.2M sodium chloride) per 1 ml of TRIreagent were added, followed by incubation for 10 min at room temperature. After centrifugation (4°C for 30 min at 5,000×g) RNA precipitated at the bottom of the tube and was washed with 75% ethanol (VWR), volume equal to TRIreagent, and centrifuged (4°C for 30 min at 5,000×g). Supernatant was removed and 1ml 75% ethanol was added. The 75% ethanol plus pellet were transferred to an RNAse free 1.5ml tube (Biopur, Eppendorf), centrifuged at room temperature at 14,000×g for 5 min and the supernatant carefully removed. The RNA pellet was air dried and resuspended in 30 ul RNAse free water. Subsequently total RNA was purified with DNAse digest using an RNeasy Mini Kit (Qiagen) according to manufacturer's instructions in RNAse-free conditions.

### RNA quantification

Quantity and purity of RNA were determined using a NanoDrop® ND-1000 Spectrophotometer (Labtech) by measuring absorbance at 260 nm and 280 nm (OD_260_, OD_280_). The instrument was blanked against water and UV measurements of 1.4 ul RNA samples were taken and the OD_260/280_ ratio and RNA concentrations (OD_260_ of 1.0 = 40 ug/ml RNA) were automatically computed using the NanoDrop® software. RNA purity was assessed by OD_260/280_ ratio, with acceptable values lying above 1.9.

### Formaldehyde agarose gel electrophoresis

RNA quality was checked by formaldehyde agarose gel electrophoresis; 1.2% agarose gel was prepared by mixing 10 ml 10× FA buffer (200 mM MOPS, 50 mM sodium acetate, 10 mM EDTA, pH 7.0) with 90 ml RNAse free water. Agarose was microwaved at high power for 1–2 min, cooled to 60°C and 1.8 ml 37% formaldehyde was added before pouring into gel cast tray. RNA samples and RNA Ladder (Sigma-Aldrich) were mixed with 2× concentrated RNA Loading Buffer containing ethidium bromide (Sigma-Aldrich) and incubated at 65°C for 5 min to denature possible secondary structures and rapidly chilled on ice. Samples were loaded and electrophoresed in 1× FA running buffer (100 ml 10× FA buffer, 20 ml 37% formaldehyde, 880 ml H_2_O) at 7 V/cm. Post-electrophoresis, gels were visualized and imaged on a UV transilluminator and digital imaging system (BioRad Laboratories).

### cDNA preparation

A master mix containing 400 ng total RNA, 25 ng/ul oligo dT primer, 4mM dNTPs and AccuScript buffer (Stratagene) to 17 ul was incubated at 65°C for 5 min and cooled to room temperature for 10 mins to allow primer annealing; DTT was added to 10mM and AccuScript Reverse Transcriptase (Stratagene) was added according to the manufacturer's protocol to a final volume of 20 ul; negative control reactions were also prepared where the addition of reverse transcriptase was omitted. The reaction mix was incubated at 42°C for 45 min to produce cDNA. Aliquots were diluted 1∶10 for long term storage.

### Reverse transcription - Polymerase Chain Reaction (RT-PCR)

All the reactions were performed on a DNA Engine Tetrad 2 Peltier Thermal Cycler (BioRad Laboratories). A list of primers used in different applications is described in **Table S2**. PCRs were set up with 10 times diluted cDNAs prepared as described above, the amount of cDNA was normalized to the ng of RNA used in Reverse Transcriptase reaction. Conditions used were annealing temperature of 58°C for 30s, and elongation at 68°C for 3 minutes over 35 cycles using Pfu High Fidelity Polymerase (Stratagene) according to manufacturer's instructions. For multiplex reactions a Multiplex PCR Kit (Qiagen) was used according to manufacturer's instructions with an annealing temperature of 60°C for 90 sec, elongation at 72°C for 1 min over 35 cycles. PCR products were purified using QIAquick PCR purification kits (Qiagen) according to manufacturer's instructions. PCR products were visualized by electrophoresis on agarose gels in 1 × TBE buffer (National Diagnostics) according to standard protocols. The quality of cDNA obtained was checked by amplifying a 780bp product from the mouse *Gapdh* gene which is ubiquitously expressed (**Figure S3**).

### Subcloning cDNAs

After amplification PCR products were subcloned into the pCR4-TOPO® vector (Invitrogen) using a TOPO TA kit (Invitrogen) according to manufacturer's instructions.

### Sequencing

Automated fluorescence sequencing was carried out with a BigDye Terminator Ready Reaction Kit (Applied Biosystems) on a 3130XL Genetic Analyser (Applied Biosystems) according to manufacturer's protocols.

### Bioinformatics analysis

We searched the UCSC Genome Browser which is developed and maintained by the Genome Bioinformatics Group within the Center for Biomolecular Science and Engineering at the University of California Santa Cruz (UCSC); the 2009 update describes 46 genome assemblies for including those for human (assembly hg19, October 2009), mouse (assembly mm9, July 2007), rat (assembly rn4, November 2004, EST database update October 2008), with extensive comparative genomics tools (Kuhn et al. 2009). Transcripts assigned as permanent have an ‘uc’ accession number. The mouse transcripts of *Dync1i1* and *Dync1i2* were also compared with entries found in the Ensembl Genome Browser release 57 (3 March 2010). ESTs mentioned in the text were those which extend from exon 1 sufficiently far into the cDNA (Dync1i1 or Dync1i2) to determine which isoform they represented.

We used several tracks within the UCSC Genome Browser to help identify possible splice variants in mouse including:

#### UCSC ‘Known Genes’

All UCSC Gene tracks show gene predictions based on data from RefSeq (NCBI RNA reference sequences), GenBank, CCDS (Consensus Coding DNA Sequences) and UniProt, thus they are a moderately conservative set of predictions, requiring the support of one GenBank RNA sequence plus at least one additional line of evidence (e.g. a UniProt, ‘Exoniphy’ prediction, or two or more expressed sequence tags (ESTs)). The RefSeq RNAs do not require additional evidence. Thus tracks include both protein-coding and putative non-coding transcripts, and compared to RefSeq genes contain more splice variants.

#### Database of Transcription Start Sites

DBTSS (http://dbtss.hgc.jp/, release 6.0.1) is a database of transcriptional start sites (TSS), based on an unique collection of precise, experimentally-determined 5′-end sequences of full-length cDNAs [26]. The use of an oligo-capping technique allows TSS to be assigned to independent genomic positions to which the first bases of sequenced cDNA were mapped, thus most cDNA sequence 5′ ends should correspond to active TSSs [43].

#### Transcription Factor Binding Sites

The HMR (Human, Mouse, Rat) conserved transcription factor binding sites track in the UCSC Genome Browser contains the location and score of transcription factor binding sites conserved in the human/mouse/rat alignment. A binding site is considered to be conserved if its score meets the threshold score for its binding matrix in all 3 species. The score and threshold are computed with the Transfac Matrix Database (v7.0) created by Biobase (http://www.gene-regulation.com/pub/databases.html). The data are purely computational, and as such not all binding sites listed are biologically functional. The analysis presented here was only undertaken for sites upstream to the transcription start (exon 1) for both *Dync1i1* and *Dync1i2*.

#### Target Scan

The Target Scan miRNA regulatory sites track of the UCSC Genome Browser shows conserved mammalian microRNA regulatory target sites in the 3′ UTR regions of Refseq Genes, as predicted by TargetScan [44]. Putative miRNA binding sites in UTRs were identified using seven-nucleotide seed regions from all known miRNA families conserved among human, mouse, rat, dog and sometimes chicken [45].

#### Protein sequence analysis

Protein sequences were aligned using ClustalW2 (http://srs.ebi.ac.uk) with Gonnet set as a substitution matrix. Subsequently they were compared using AliSubSimP (http://srs.ebi.ac.uk) with EBLOSUM62 as a scoring matrix.

## Supporting Information

Figure S1 Example of amplifying *Dync1i1.C* in non-neuronal mouse tissues. Primers DIC1 Ex1 for and DIC1_R rev amplify all six *Dync1i1* isoforms (see Figure 2), however in non-neuronal tissues we detect isoform *Dync1i1.C* only (420bp) in ovary and testis. ‘+’ lanes are cDNA, ‘−’ lanes control for genomic DNA contamination and have no reverse transcriptase.
(0.86 MB TIF)

Figure S2 Amplification of *Dync1i1.F* in mouse neuronal tissues. Primers DIC1_1.1 for and DIC1_iso14 rev amplify a 155 bp product from isoform 1.F. One sample in hippocampus and one in cortex failed to amplify. ‘+’ lanes are cDNA, ‘−’ lanes control for genomic DNA contamination and have no reverse transcriptase.
(3.82 MB TIF)

Figure S3 Example of *Gapdh* amplicon in mouse tissues. *Gapdh* is ubiquitously expressed and a single band of 780 bp was visualised in ‘reverse transcriptase positive’ samples while no bands were visible in samples in which no reverse transcriptase had been added. This was our control for cDNA quality.
(2.47 MB TIF)

Table S1 Exon intron boundaries of mouse *Dync1i1* and *Dync1i2*.
(0.02 MB DOCX)

Table S2 Primer sequences used to determine the splicing pattern of *Dync1i1* and *Dync1i2*.
(0.01 MB DOCX)

Table S3 The position of protein domains in the longest DYNC1I1 and DYNC1I2 isoforms.
(0.01 MB DOCX)
