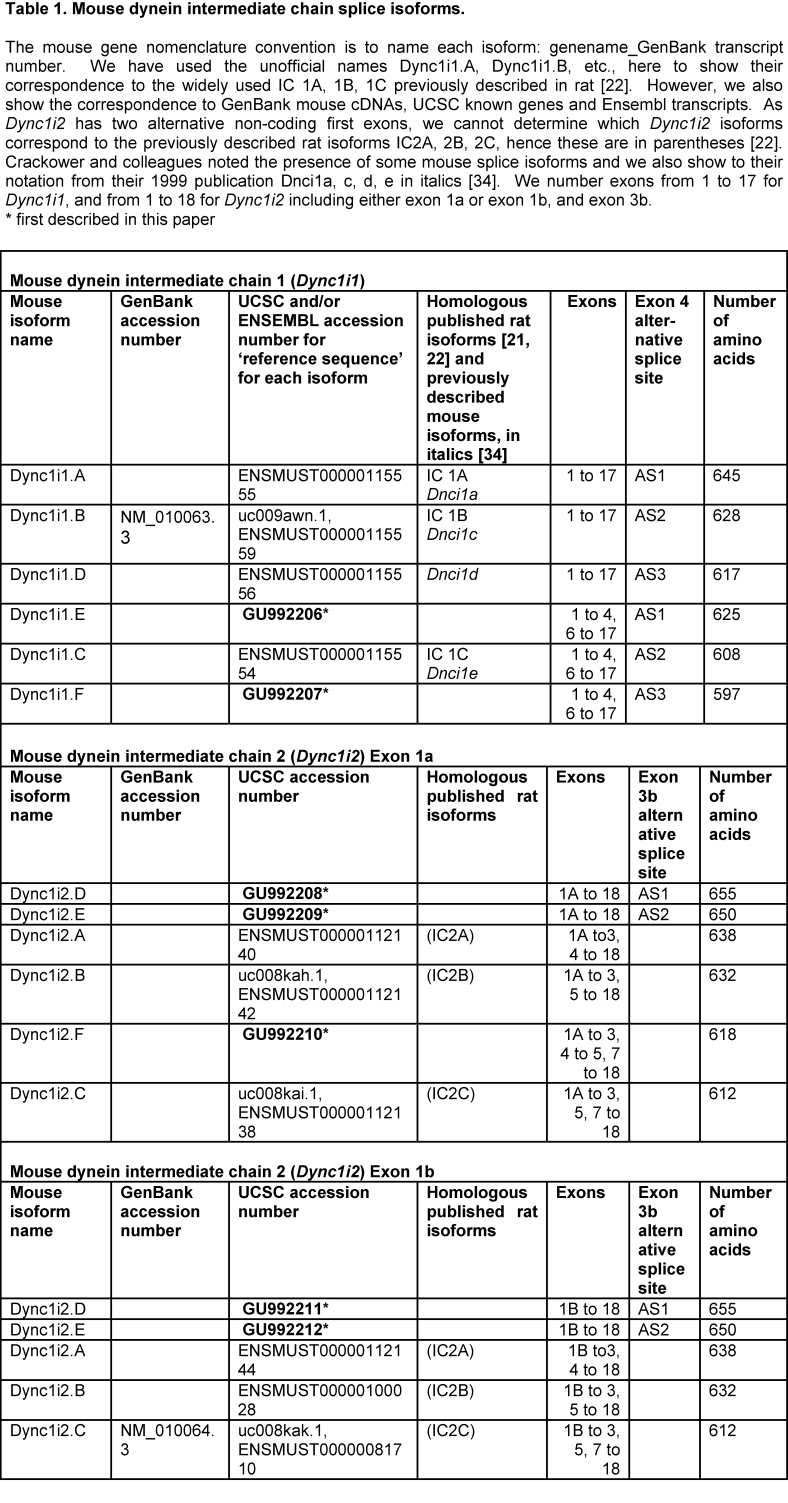# Correction: Mouse Cytoplasmic Dynein Intermediate Chains: Identification of New Isoforms, Alternative Splicing and Tissue Distribution of Transcripts

**DOI:** 10.1371/annotation/59badad8-6e55-46f8-8bf1-7a8a957bc68e

**Published:** 2010-10-08

**Authors:** Anna Kuta, Wenhan Deng, Ali Morsi El-Kadi, Gareth T. Banks, Majid Hafezparast, K. Kevin Pfister, Elizabeth M. C. Fisher

Table 1 one is incomplete. See the correct version here: 

**Figure:**