# Supplementary figures and images for: Mouse Cytoplasmic Dynein Intermediate Chains: Identification of New Isoforms, Alternative Splicing and Tissue Distribution of Transcripts
Source: PLoS One. 2010 Jul 21;5(7):e11682. doi: 10.1371/journal.pone.0011682 (PMC2908135; doi:10.1371/journal.pone.0011682)

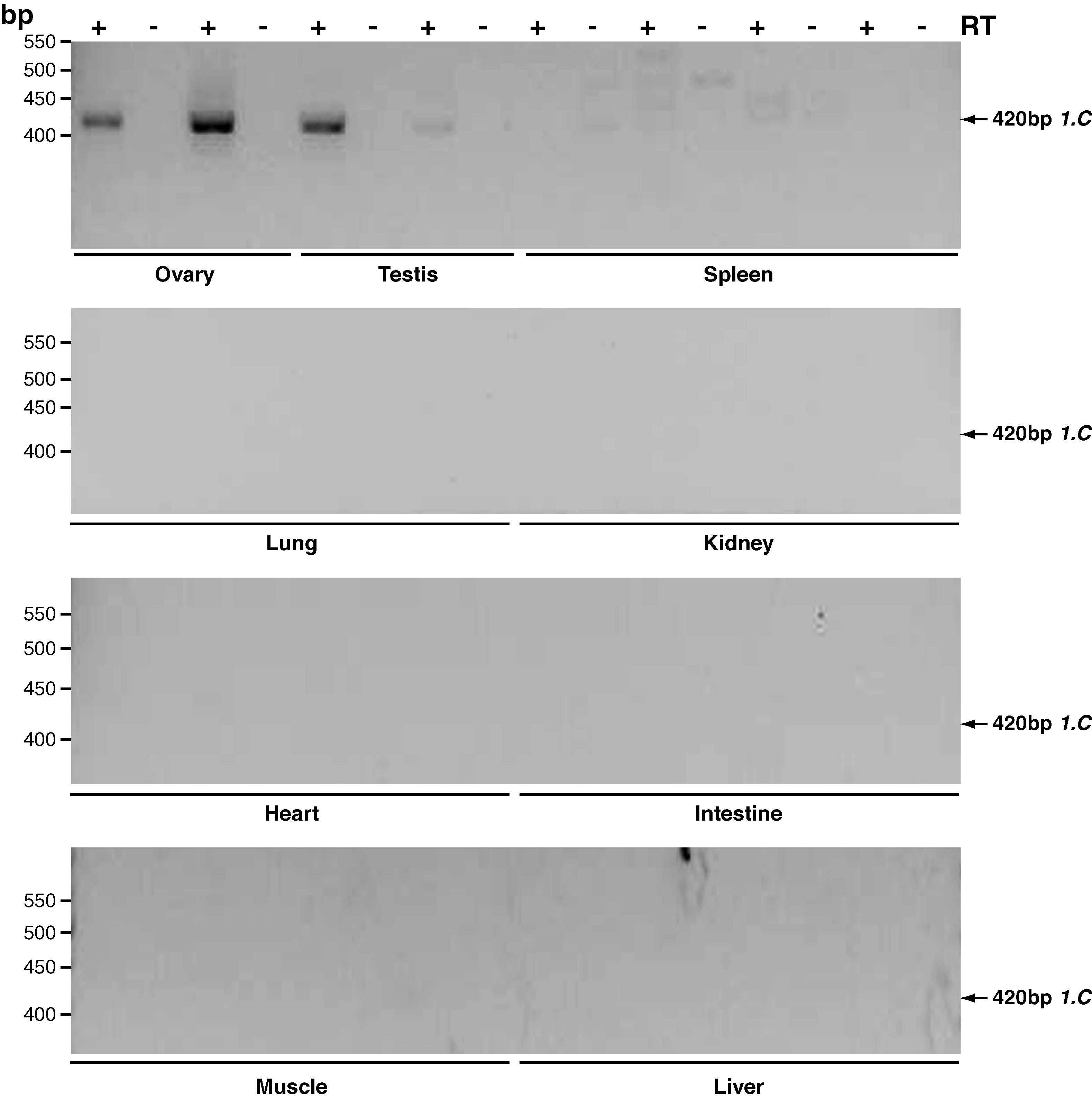

Supplement: Figure S1 — Example of amplifying Dync1i1.C in non-neuronal mouse tissues. Primers DIC1 Ex1 for and DIC1_R rev amplify all six Dync1i1 isoforms (see Figure 2), however in non-neuronal tissues we detect isoform Dync1i1.C only (420bp) in ovary and testis. ‘+’ lanes are cDNA, ‘−’ lanes control for genomic DNA contamination and have no reverse transcriptase. (0.86 MB TIF) [file pone.0011682.s001.tif]

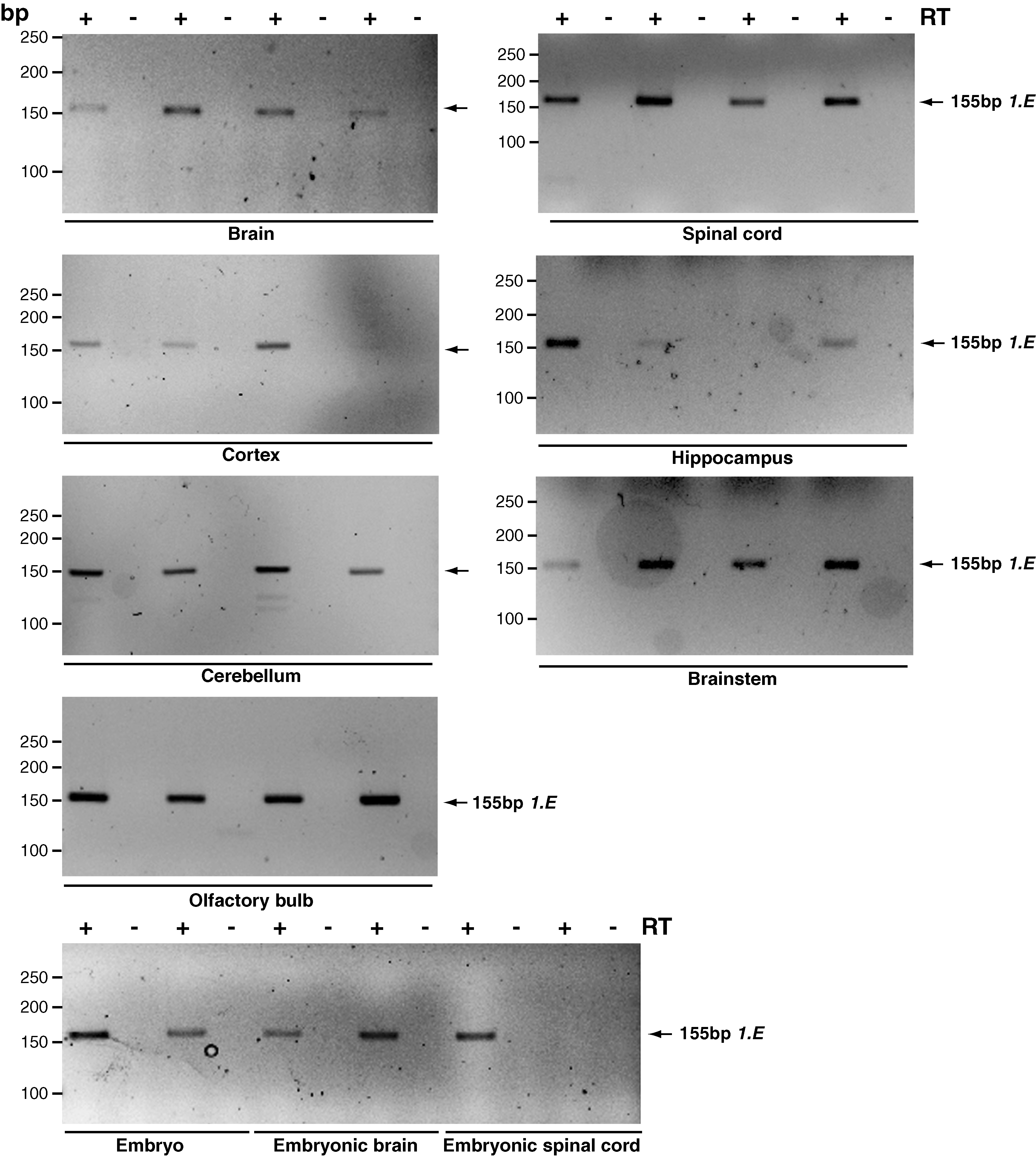

Supplement: Figure S2 — Amplification of Dync1i1.F in mouse neuronal tissues. Primers DIC1_1.1 for and DIC1_iso14 rev amplify a 155 bp product from isoform 1.F. One sample in hippocampus and one in cortex failed to amplify. ‘+’ lanes are cDNA, ‘−’ lanes control for genomic DNA contamination and have no reverse transcriptase. (3.82 MB TIF) [file pone.0011682.s002.tif]

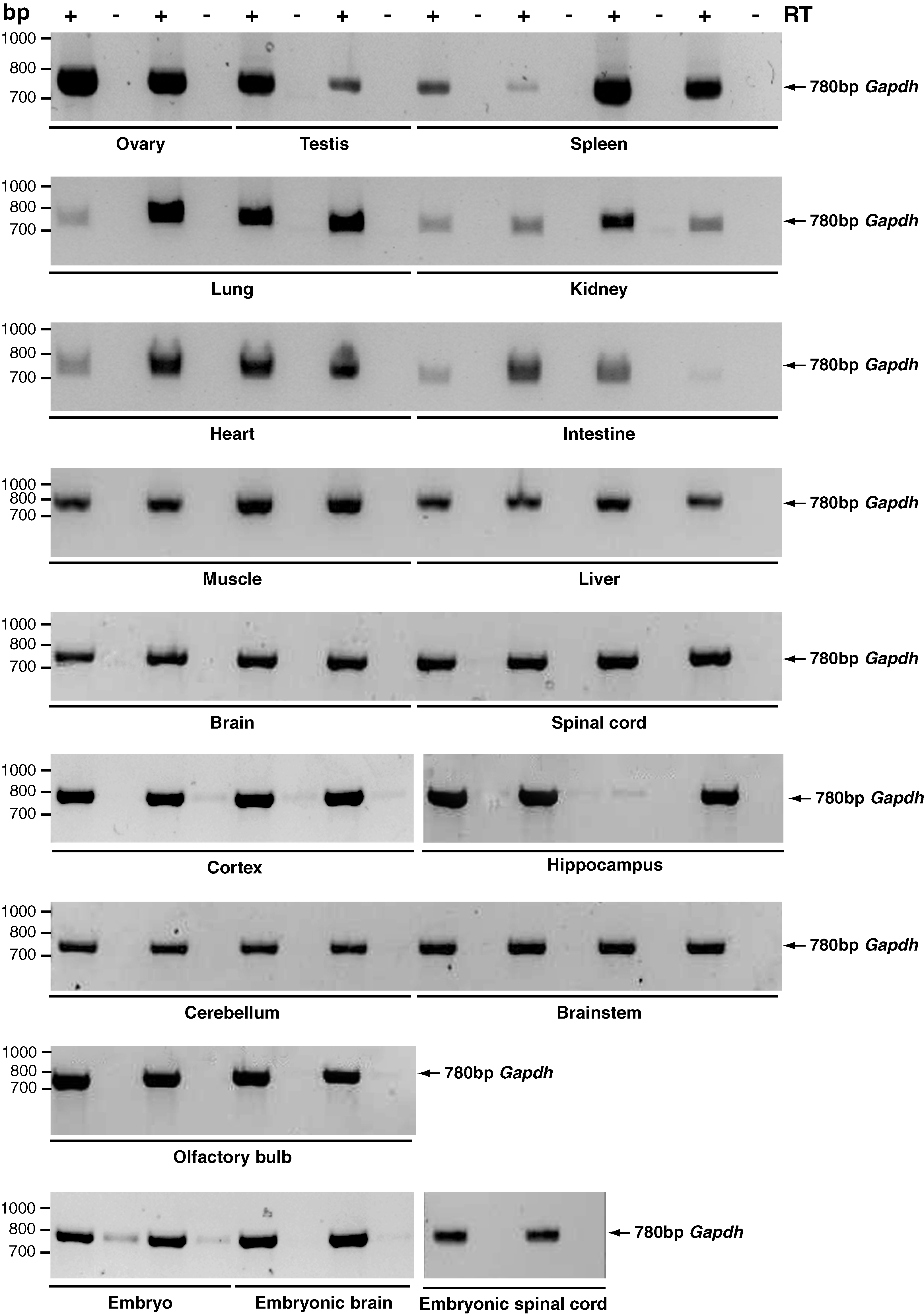

Supplement: Figure S3 — Example of Gapdh amplicon in mouse tissues. Gapdh is ubiquitously expressed and a single band of 780 bp was visualised in ‘reverse transcriptase positive’ samples while no bands were visible in samples in which no reverse transcriptase had been added. This was our control for cDNA quality. (2.47 MB TIF) [file pone.0011682.s003.tif]
