# Supplementary material for: Mouse Cytoplasmic Dynein Intermediate Chains: Identification of New Isoforms, Alternative Splicing and Tissue Distribution of Transcripts
Source: PLoS One. 2010 Jul 21;5(7):e11682. doi: 10.1371/journal.pone.0011682 (PMC2908135; doi:10.1371/journal.pone.0011682)
Supplement: Table S1 — Exon intron boundaries of mouse Dync1i1 and Dync1i2. (0.02 MB DOCX) [file pone.0011682.s004.docx]

**Table S1. Exon intron boundaries of mouse *Dync1i1* and *Dync1i2*.**

Taken from Ensembl mouse genomic sequence release 57.

| ***Dync1i1***  The 5’ and 3’ ends are not well resolved, here we base the gene boundaries based on the Ensembl curated transcript ENSMUST00000115555. | | | |
| --- | --- | --- | --- |
| **Exon** | **Intron** | **Size bp** | **start – finish bp** |
| **1** |  | 174 | 5,675,763 – 5,675,936 |
|  | **1** | 31,441 | 5,675937 – 5,707,377 |
| **2** |  | 117 | 5,707,378 – 5,707,494 |
|  | **2** | 9,535 | 5,707,495 – 5,717,029 - |
| **3** |  | 115 | 5,717,030 – 5,717,144 |
|  | **3** | 2,505 | 5,717,145 – 5,719,649 - |
| **4** |  | 142 | 5,719,650 – 5,719,791 |
|  | **4** | 14,647 | 5,719,792 – 5,734,438 - |
| **5** |  | 60 | 5,734,439 – 5,734,498 |
|  | **5** | 16,276 | 5,734,499 – 5,750,774 - |
| **6** |  | 116 | 5,750,775 – 5,750,890 |
|  | **6** | 104,214 | 5,750,891 – 5,855,104 - |
| **7** |  | 90 | 5,855,105 – 5,855,194 |
|  | **7** | 8,121 | 5,855,195 – 5,863,315 - |
| **8** |  | 163 | 5,863,316 – 5,863,478 |
|  | **8** | 2,351 | 5,863,479 – 5,865,829 - |
| **9** |  | 100 | 5,865,830 – 5,865,929 |
|  | **9** | 7,235 | 5,865,930 – 5,873,164 - |
| **10** |  | 126 | 5,873,165 – 5,873,290 |
|  | **10** | 38,550 | 5,873,291 – 5,911,840 - |
| **11** |  | 147 | 5,911,841 – 5,911,987 |
|  | **11** | 4,715 | 5,911,988 – 5,916,702 - |
| **12** |  | 114 | 5,916,703 – 5,916,816 |
|  | **12** | 2,557 | 5,916,817 – 5,919,373 - - |
| **13** |  | 134 | 5,919,374 – 5,919,507 |
|  | **13** | 2,557 | 5,919,508 – 5,922,064 - |
| **14** |  | 145 | 5,922,065 – 5,922,209 |
|  | **14** | 34,060 | 5,922,210 – 5,956,269 |
| **15** |  | 141 | 5,956,270 – 5,956,410 |
|  | **15** | 4,044 | 5,956,411 – 5,960,454 |
| **16** |  | 126 | 5,960,455 – 5,960,580 |
|  | **16** | 16,791 | 5,960,581 – 5,977,371 |
| **17** |  | 659 | 5,977,372 – 5,978,030 |
| ***Dync1i2***  The 5’ end of the Exons 1a and 1b are not well resolved, here we base the 1a and 1b exon sizes on two Ensembl curated transcripts, ENSMUST00000112140 (contains exon 1a) and ENSMUST00000112144 (contains exon 1b). | | | |
| **1a** |  | 105 | **71,049,798 – 71,049,902** |
|  | **1a** | 125 | **71,049,903 – 71,050,028** |
| **1b** |  | 103 | **71,050,029 – 71,050,131** |
|  | **1b** | 2,364 | **71,050,132 – 71,052,495** |
| **2** |  | 117 | 71,052,496 – 71,052,612 |
|  | **2** | 4,745 | 71,052,613 – 71,057,357 |
| **3** |  | 118 | 71,057,358 – 71,057,475 |
|  | **3** | 3,711 | 71,057,476 – 71,061,187 |
| **3b** |  | 51 | 71,061,188 – 71,061,238 |
|  | **3b** | 4,644 | 71,061,239 – 71,065,883 |
| **4** |  | 18 | 71,065,884 – 71,065,901 |
|  | **4** | 755 | 71,065,902 – 71,066,656 - |
| **5** |  | 91 | 71,066,657 – 71,066,747 |
|  | **5** | 4,958 | 71,066,748 – 71,071,705 |
| **6** |  | 60 | 71,071,706 – 71,071,765 |
|  | **6** | 2,206 | 71,071,766 – 71,073,971 |
| **7** |  | 116 | 71,073,972 – 71,074,087 |
|  | **7** | 10,658 | 71,074,088 – 71,084,745 |
| **8** |  | 96 | 71,084,746 – 71,084,841 |
|  | **8** | 228 | 71,084,842 – 71,085,069 |
| **9** |  | 163 | 71,085,070 – 71,085,232 |
|  | **9** | 113 | 71,085,233 – 71,085,345 |
| **10** |  | 100 | 71,085,346 – 71,085,445 |
|  | **10** | 432 | 71,085,446 – 71,085,877 |
| **11** |  | 126 | 71,085,878 – 71,086,003 |
|  | **11** | 840 | 71,086,004 – 71,086,843 |
| **12** |  | 147 | 71,086,844 – 71,086,990 |
|  | **12** | 375 | 71,086,991 – 71,087,365 |
| **13** |  | 114 | 71,087,366 – 71,087,479 |
|  | **13** | 296 | 71,087,480 – 71,087,775 |
| **14** |  | 134 | 71,087,776 – 71,087,909 |
|  | **14** | 989 | 71,087,910 – 71,088,898 |
| **15** |  | 145 | 71,088,899 – 71,089,043 |
|  | **15** | 7,097 | 71,089,044 – 71,096,140 - |
| **16** |  | 141 | 71,096,141 – 71,096,281 |
|  | **16** | 2,597 | 71,096,282 – 71,098,878 - |
| **17** |  | 126 | 71,098,879 – 71,099,004 |
|  | **17** | 1,700 | 71,099,005 – 71,100,704 |
| **18** |  | 656 | 71,100,705 – 71,101,360 |
