# Supplementary material for: Mouse Cytoplasmic Dynein Intermediate Chains: Identification of New Isoforms, Alternative Splicing and Tissue Distribution of Transcripts
Source: PLoS One. 2010 Jul 21;5(7):e11682. doi: 10.1371/journal.pone.0011682 (PMC2908135; doi:10.1371/journal.pone.0011682)
Supplement: Table S2 — Primer sequences used to determine the splicing pattern of Dync1i1 and Dync1i2. (0.01 MB DOCX) [file pone.0011682.s005.docx]

**Table S2. Primer sequences used to determine the splicing pattern of *Dync1i1* and *Dync1i2*.**

| **Application** | **Mouse gene** | **Primer name** | **Primer sequence (5’ to 3’)** |
| --- | --- | --- | --- |
| PCR | *Dync1i1* | DIC1_Ex 1 for  DIC1_Ex 17 rev  DIC1_1.1 for  DIC1_AS4 rev  DIC1_5 rev  DIC1_iso14 rev  DIC1_R rev | CTCCACGACCTCCAGTGGAG  ACGCACATGCTCTAAGATCG  CTCTAGTGCAGCCGTTGCAT  GGGAGTGCTCACTGATTTCG  AAGTTCTGAGTCTGACTGCAG  GCAGTCGTCTCCTTGTTAATGG  CTAGGCAGGAAATCCACCTG |
|  | *Dync1i2* | DIC2_Ex1a for  DIC2_Ex1b for  DIC2_Ex18 rev  DIC2_N4 rev  DIC2_6 rev  DIC2_iso24 rev  DIC2_2.1 rev  DIC2_R rev | GGCCGGTGTATCTGTTTCAAC  CAGTTGGAGAGGGACGTTC  AGAAGGGAAATGGCATCAAC  GAGGGACCCAGTACTCAG  AAATCGGAATCTGAGTGAAGC  CAAGTTTAATAGGTCCTCGTCTA  AACTTGATTGAAGCTAGAATCCTC  CTCGAGGGGGAAAGTCAA C |
|  | *Gapdh* | Gapdh for  Gapdh rev | ACTCCACTCACGGCAAATTC  ATGTAGGCCATGAGGTCCAC |
| Sequencing | *Dync1i1* | DIC1_Ex 1 for  DIC1_Ex 3 rev  DIC1_Ex 9 for  DIC1_Ex 10 rev  DIC1_Ex 16 for | CTCCACGACCTCCAGTGGAG  CTCCGGTGATATGCCAATGC  TGAACATTGGTCTAAGCATCGG  TGGAAGACATATTCTGGTGTGG  AGGAGCATCTGCCCTAAAC |
|  | *Dync1i2* | DIC2_Ex1a for  DIC2_Ex1b for  DIC2_Ex9 for  DIC2_Ex14 for | GGCCGGTGTATCTGTTTCAAC  CAGTTGGAGAGGGACGTTC  CGAGAATTGTAGAAAGAGCC  GTTGTGGGCAGTGAAGAAG |

‘Ex’ refers to the number of the exon containing the primer binding site e.g. Ex 1 is exon 1.
