# Supplementary material for: Mouse Cytoplasmic Dynein Intermediate Chains: Identification of New Isoforms, Alternative Splicing and Tissue Distribution of Transcripts
Source: PLoS One. 2010 Jul 21;5(7):e11682. doi: 10.1371/journal.pone.0011682 (PMC2908135; doi:10.1371/journal.pone.0011682)
Supplement: Table S3 — The position of protein domains in the longest DYNC1I1 and DYNC1I2 isoforms. (0.01 MB DOCX) [file pone.0011682.s006.docx]

**Table S3. The position of protein domains in the longest DYNC1I1 and DYNC1I2 isoforms.**

| **Structural feature/domain** | **DYNC1I1.A (amino acid)** | **DYNC1I2.D (amino acid)** |
| --- | --- | --- |
| Coiled coil | 1 – 59 | 1 – 60 |
| DYNLT binding | 148 – 160 | 155 – 167 |
| DYNLL binding | 167 – 173 | 174 – 180 |
| Dimerisation | 187 – 244 | 194 – 253 |
| DYNLRB | 245 – 284 | 254 – 293 |
| WD40 repeats 1  2  3  4  5  6  7 | 285 – 326  338 – 370  388 – 420  433 – 470  482 – 514  530 – 562  575 - 607 | 294 – 335  347 – 379  396 – 429  441 – 479  491 – 523  539 – 571  584 – 616 |
